# Supplementary material for: Variation in the availability and cost of essential medicines for non-communicable diseases in Uganda: A descriptive time series analysis
Source: PLoS One. 2020 Dec 23;15(12):e0241555. doi: 10.1371/journal.pone.0241555 (PMC7757794; doi:10.1371/journal.pone.0241555)
Supplement: S1 Fig — These time series plots show the week-by-week shifts in the proportion of facilities stocking specific essential medicines, broken down by sector. Each colored line represents one essential medicine; movement on the y axis from week to week indicates change in the proportion of facilities for which that medicine was available. (PDF) [file pone.0241555.s001.pdf]

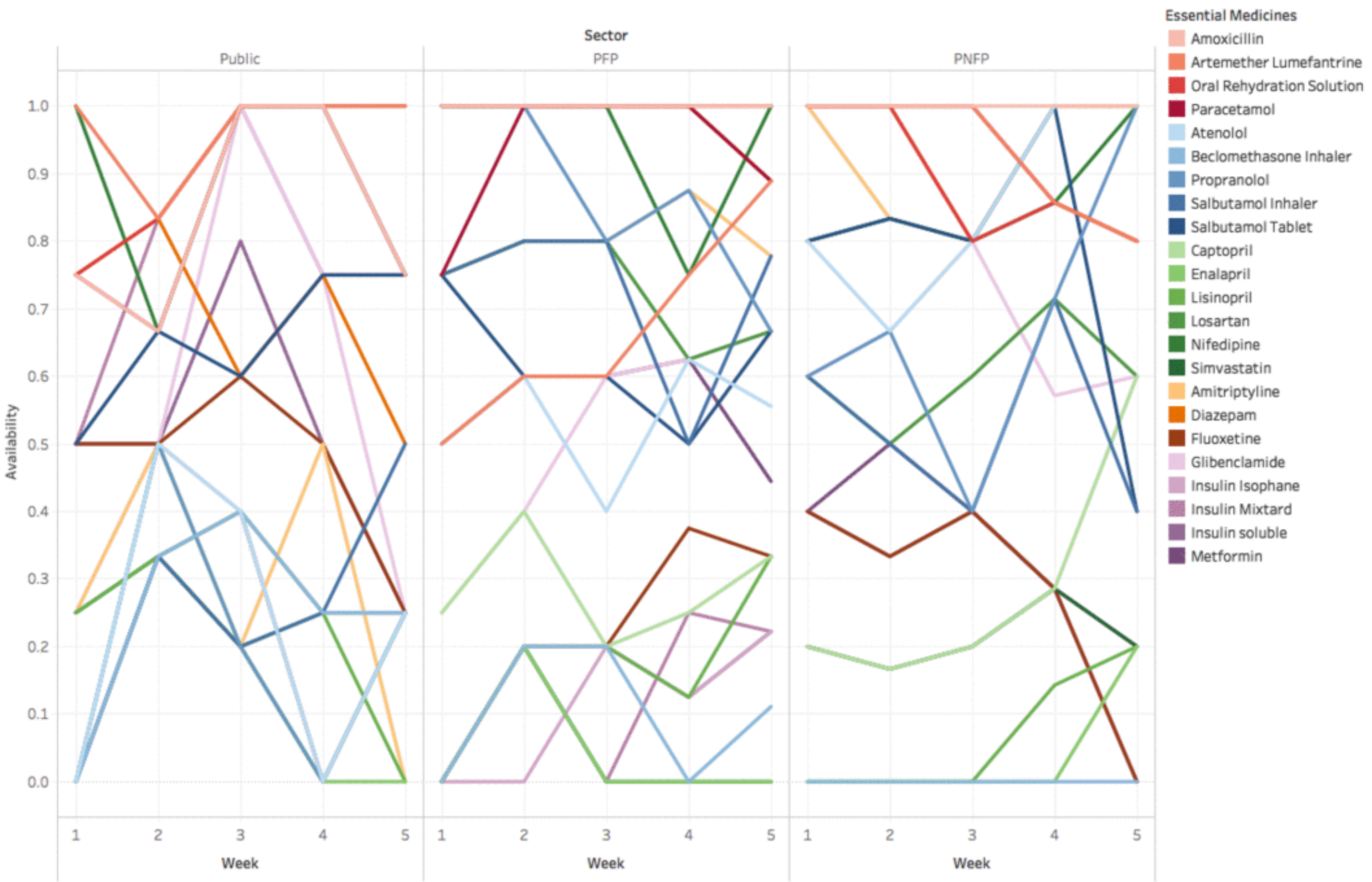

Sector
Public
PFP
PNFP
Availability
1.0
0.9
0.8
0.7
0.6
0.5
0.4
0.3
0.2
0.1
0.0
1
2
3
4
5
Week
Essential Medicines
Amoxicillin
Artemether Lumefantrine
Oral Rehydration Solution
Paracetamol
Atenolol
Beclomethasone Inhaler
Propranolol
Salbutamol Inhaler
Salbutamol Tablet
Captopril
Enalapril
Lisinopril
Losartan
Nifedipine
Simvastatin
Amitriptyline
Diazepam
Fluoxetine
Glibenclamide
Insulin Isophane
Insulin Mixtard
Insulin soluble
Metformin

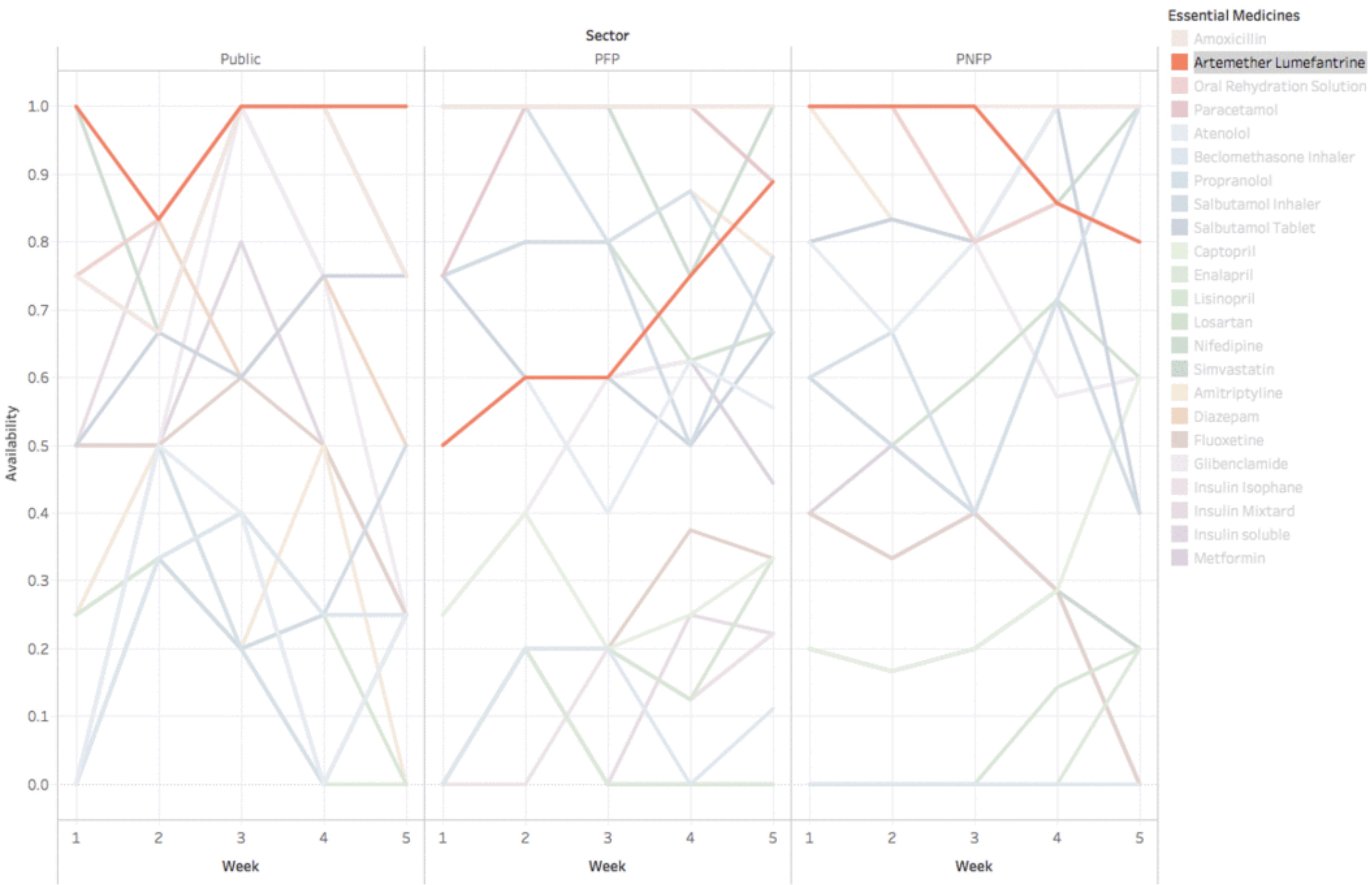

Sector
Public
PFP
PNFP
Availability
1.0
0.9
0.8
0.7
0.6
0.5
0.4
0.3
0.2
0.1
0.0
1
2
3
4
5
Week
Essential Medicines
Amoxicillin
Artemether Lumefantrine
Oral Rehydration Solution
Paracetamol
Atenolol
Beclomethasone Inhaler
Propranolol
Salbutamol Inhaler
Salbutamol Tablet
Captopril
Enalapril
Lisinopril
Losartan
Nifedipine
Simvastatin
Amitriptyline
Diazepam
Fluoxetine
Glibenclamide
Insulin Isophane
Insulin Mixtard
Insulin soluble
Metformin

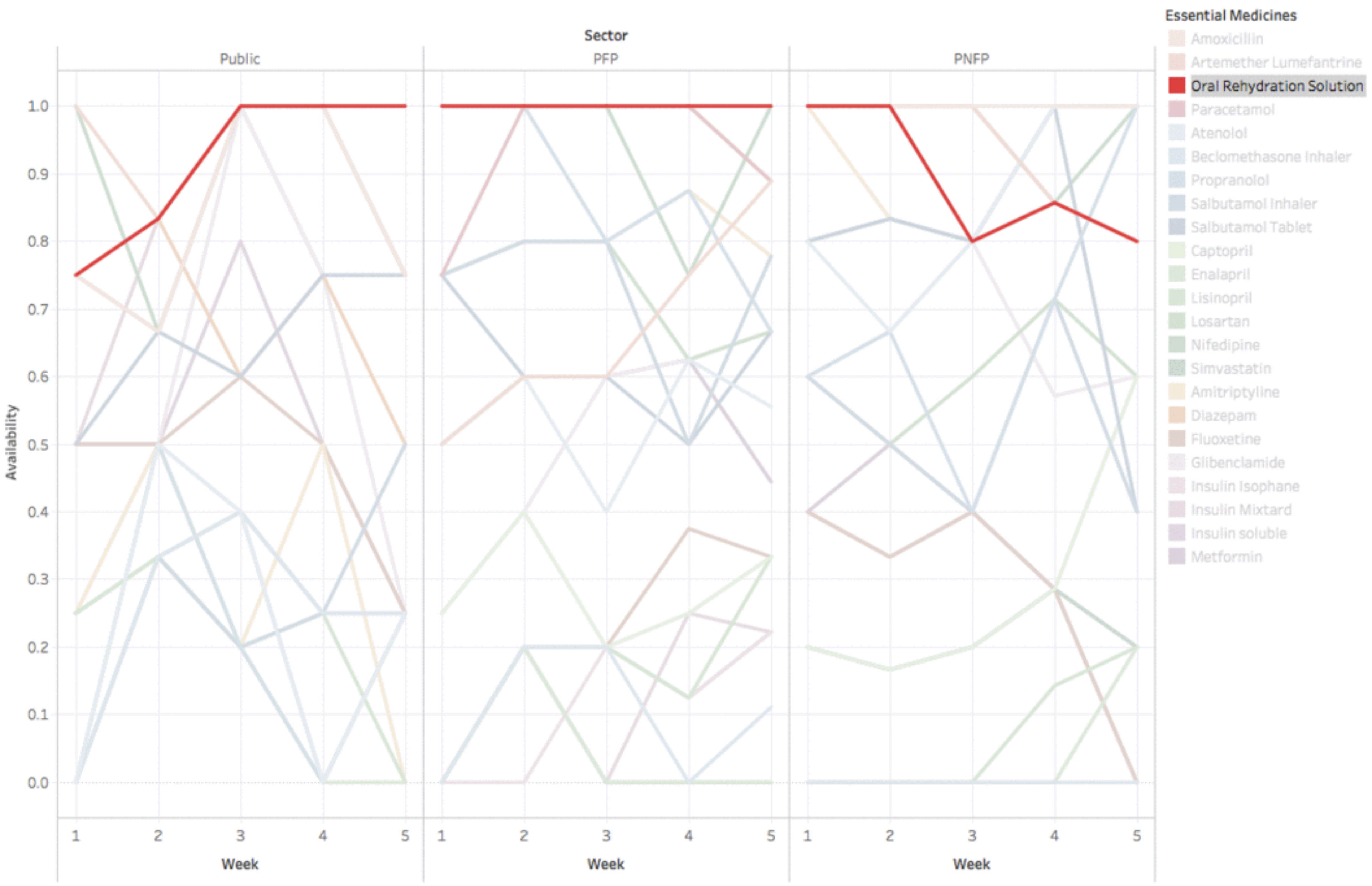

Sector
Public
PFP
PNFP
Availability
1.0
0.9
0.8
0.7
0.6
0.5
0.4
0.3
0.2
0.1
0.0
1
2
3
4
5
Week
Essential Medicines
Amoxicillin
Artemether Lumefantrine
Oral Rehydration Solution
Paracetamol
Atenolol
Beclomethasone Inhaler
Propranolol
Salbutamol Inhaler
Salbutamol Tablet
Captopril
Enalapril
Lisinopril
Losartan
Nifedipine
Simvastatin
Amitriptyline
Diazepam
Fluoxetine
Glibenclamide
Insulin Isophane
Insulin Mixtard
Insulin soluble
Metformin

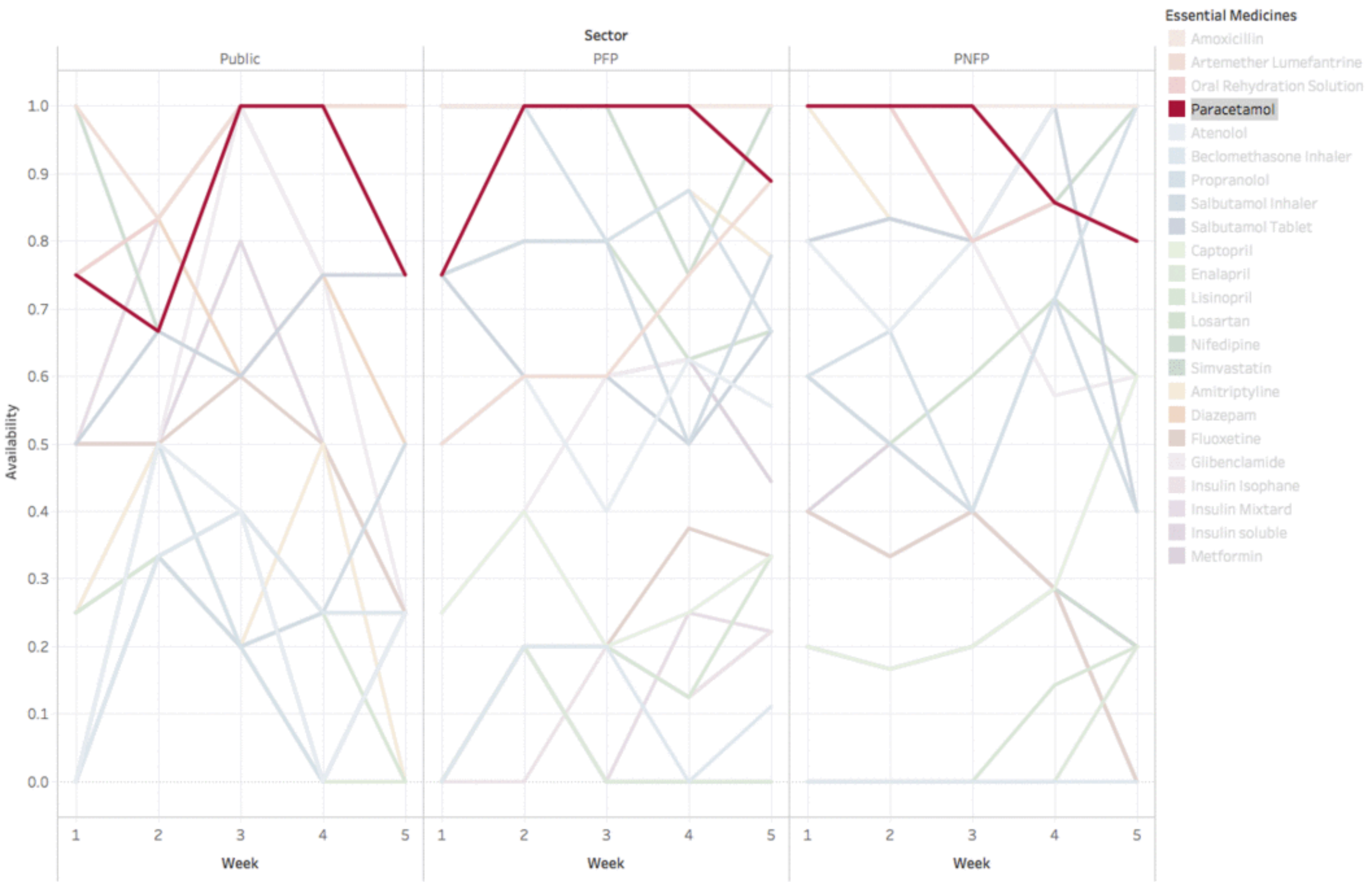
Sector
Public
PFP
PNFP
Availability
1.0
0.9
0.8
0.7
0.6
0.5
0.4
0.3
0.2
0.1
0.0
1
2
3
4
5
Week
Essential Medicines
Amoxicillin
Artemether Lumefantrine
Oral Rehydration Solution
Paracetamol
Atenolol
Beclomethasone Inhaler
Propranolol
Salbutamol Inhaler
Salbutamol Tablet
Captopril
Enalapril
Lisinopril
Losartan
Nifedipine
Simvastatin
Amitriptyline
Diazepam
Fluoxetine
Glibenclamide
Insulin Isophane
Insulin Mixtard
Insulin soluble
Metformin

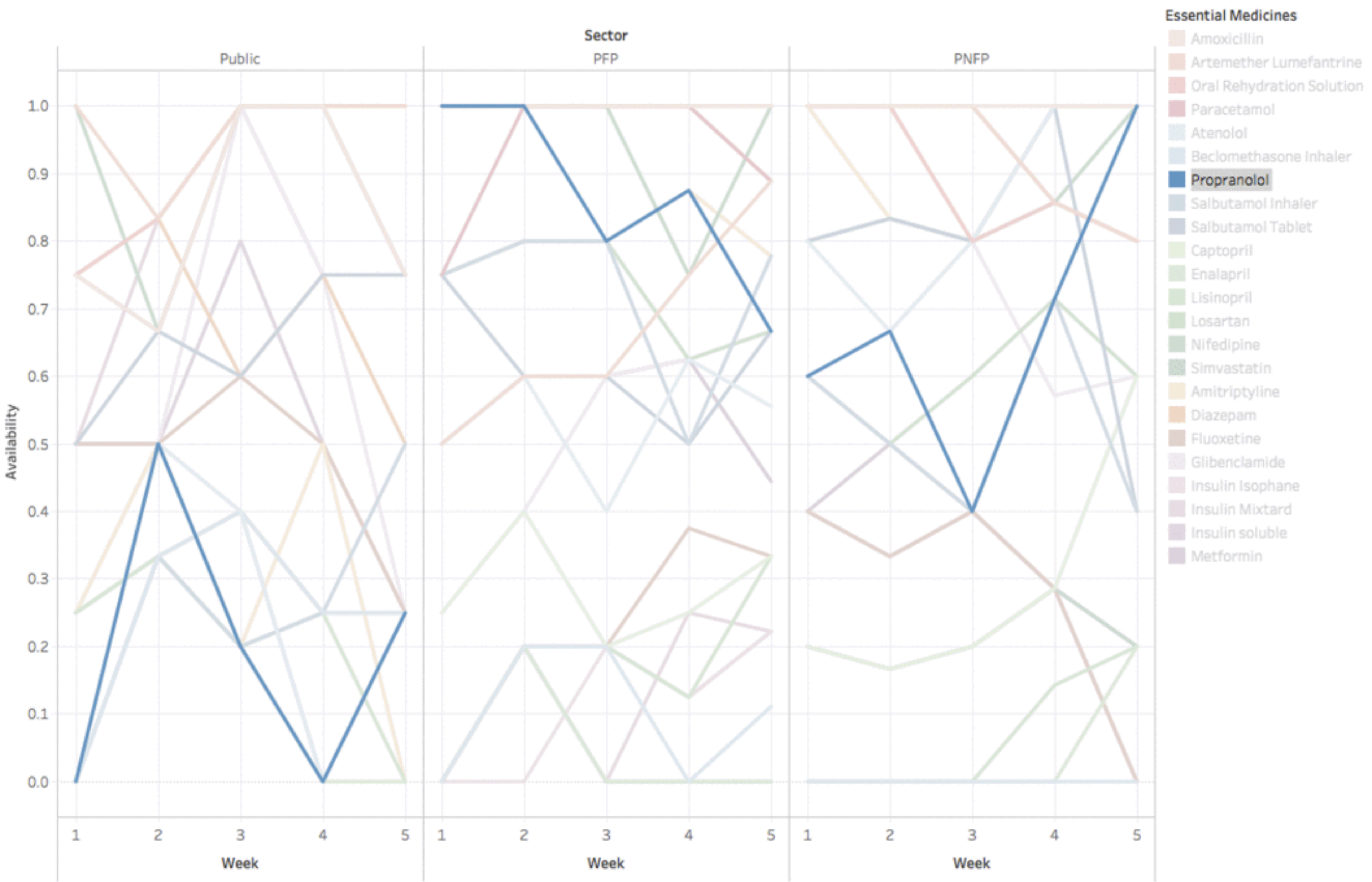
Sector
Public
PFP
PNFP
Availability
1.0
0.9
0.8
0.7
0.6
0.5
0.4
0.3
0.2
0.1
0.0
1
2
3
4
5
Week
Essential Medicines
Amoxicillin
Artemether Lumefantrine
Oral Rehydration Solution
Paracetamol
Atenolol
Beclomethasone Inhaler
Propranolol
Salbutamol Inhaler
Salbutamol Tablet
Captopril
Enalapril
Lisinopril
Losartan
Nifedipine
Simvastatin
Amitriptyline
Diazepam
Fluoxetine
Glibenclamide
Insulin Isophane
Insulin Mixtard
Insulin soluble
Metformin

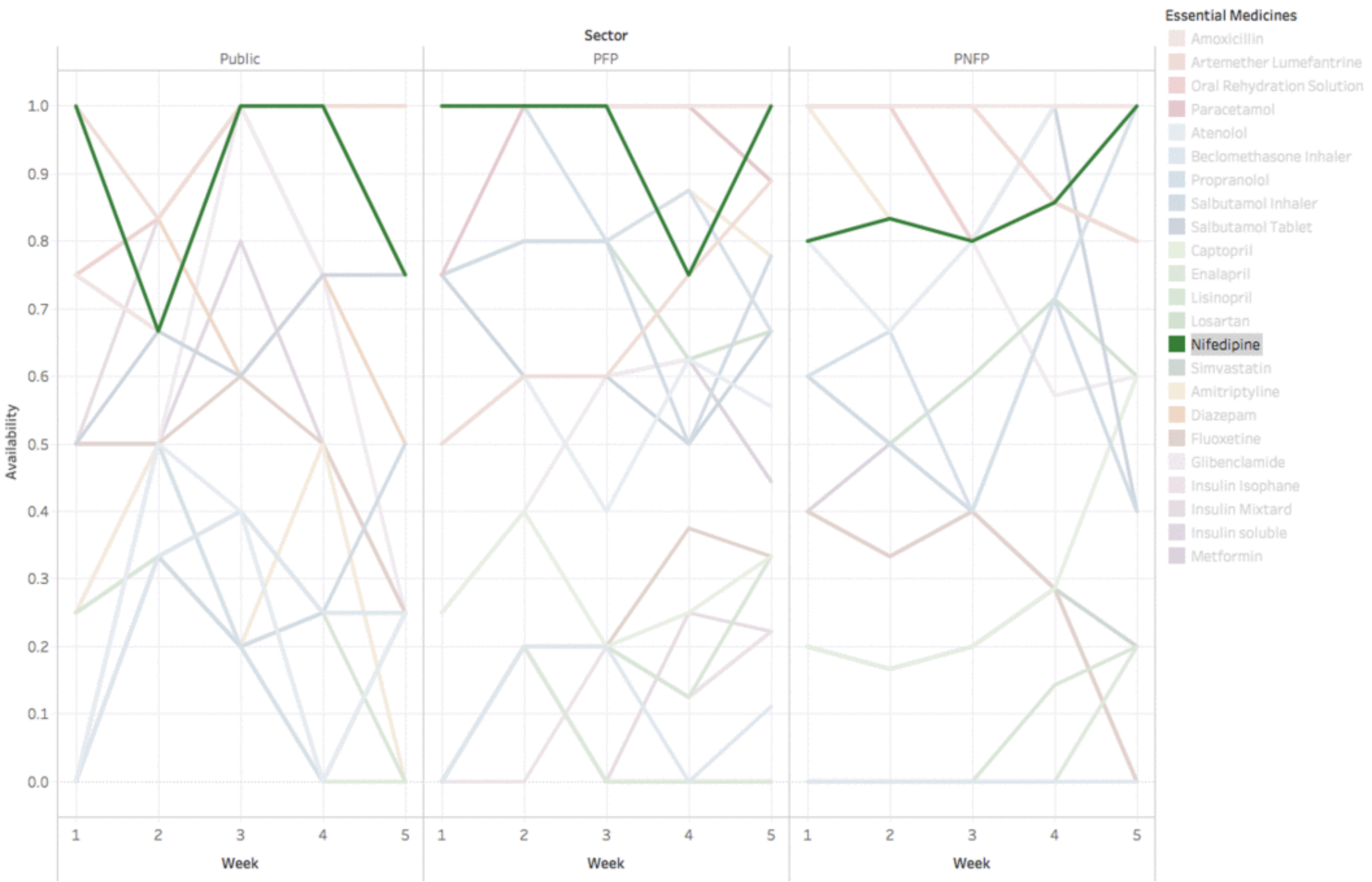

Sector
Public
PFP
PNFP
Availability
1.0
0.9
0.8
0.7
0.6
0.5
0.4
0.3
0.2
0.1
0.0
1
2
3
4
5
Week
Essential Medicines
Amoxicillin
Artemether Lumefantrine
Oral Rehydration Solution
Paracetamol
Atenolol
Beclomethasone Inhaler
Propranolol
Salbutamol Inhaler
Salbutamol Tablet
Captopril
Enalapril
Lisinopril
Losartan
Nifedipine
Simvastatin
Amitriptyline
Diazepam
Fluoxetine
Glibenclamide
Insulin Isophane
Insulin Mixtard
Insulin soluble
Metformin

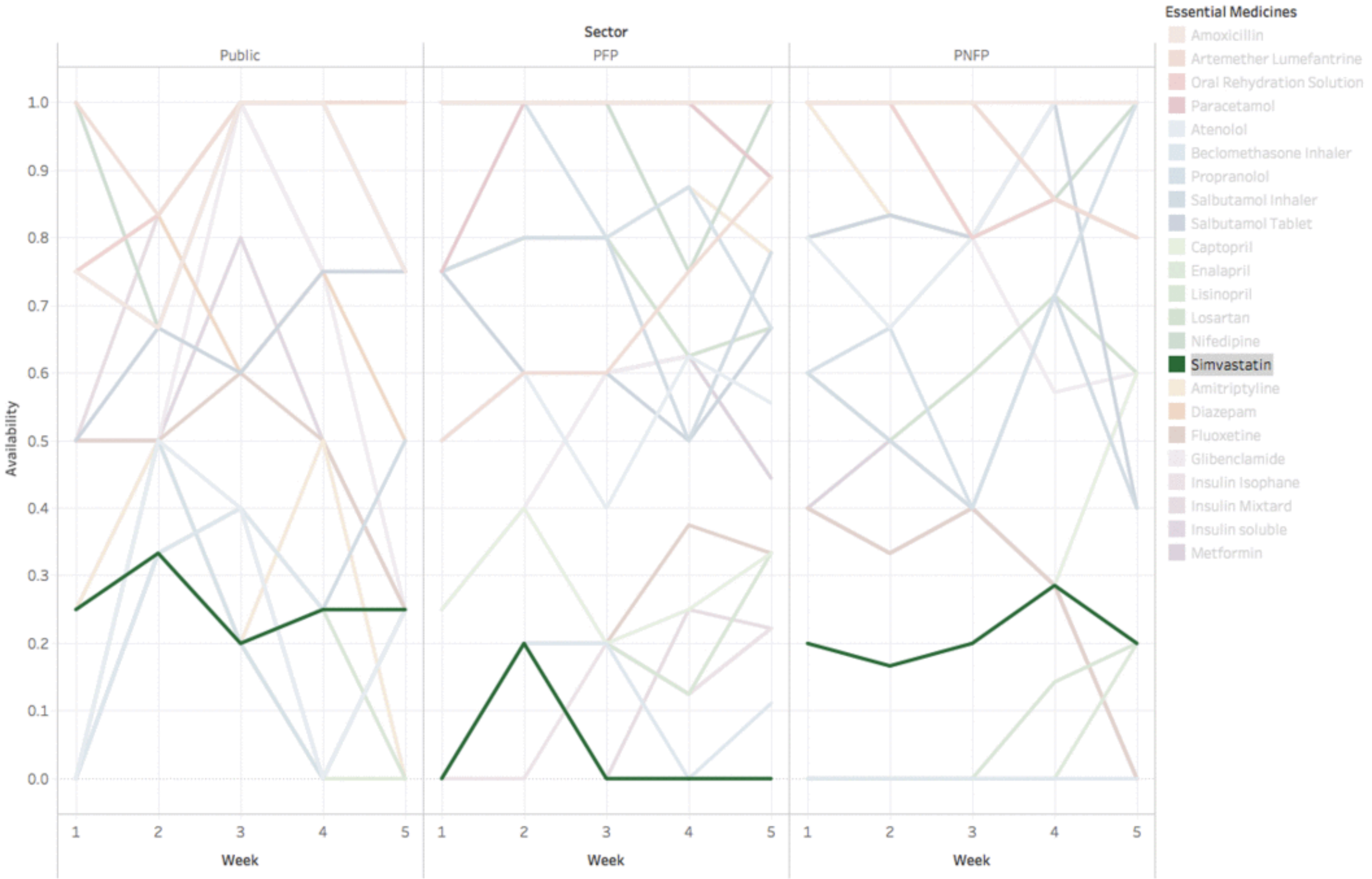

Sector
Public
PFP
PNFP
Availability
1.0
0.9
0.8
0.7
0.6
0.5
0.4
0.3
0.2
0.1
0.0
1
2
3
4
5
Week
Essential Medicines
Amoxicillin
Artemether Lumefantrine
Oral Rehydration Solution
Paracetamol
Atenolol
Beclomethasone Inhaler
Propranolol
Salbutamol Inhaler
Salbutamol Tablet
Captopril
Enalapril
Lisinopril
Losartan
Nifedipine
Simvastatin
Amitriptyline
Diazepam
Fluoxetine
Glibenclamide
Insulin Isophane
Insulin Mixtard
Insulin soluble
Metformin

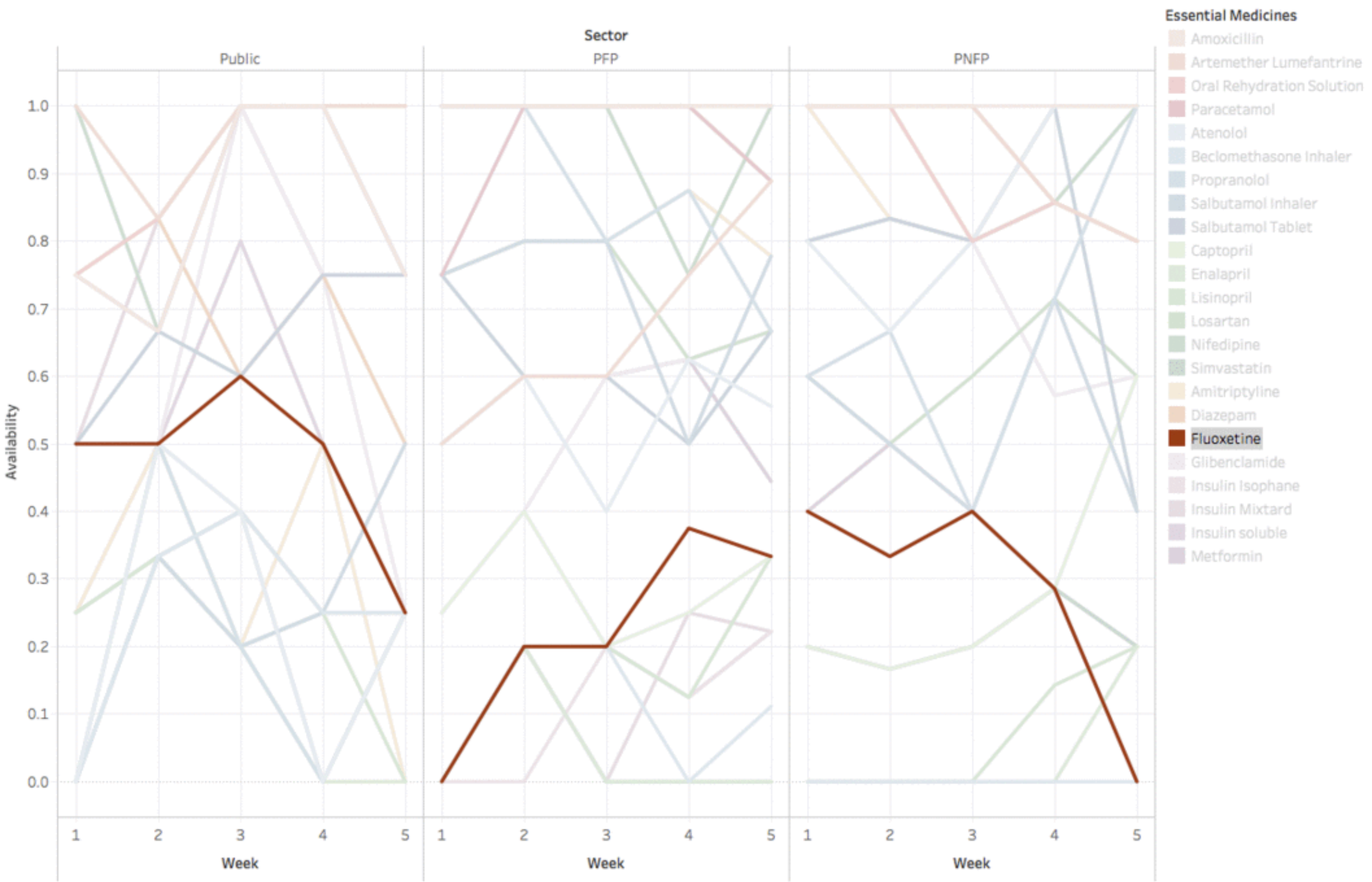
Sector
Public
PFP
PNFP
Availability
1.0
0.9
0.8
0.7
0.6
0.5
0.4
0.3
0.2
0.1
0.0
1
2
3
4
5
Week
Essential Medicines
Amoxicillin
Artemether Lumefantrine
Oral Rehydration Solution
Paracetamol
Atenolol
Beclomethasone Inhaler
Propranolol
Salbutamol Inhaler
Salbutamol Tablet
Captopril
Enalapril
Lisinopril
Losartan
Nifedipine
Simvastatin
Amitriptyline
Diazepam
Fluoxetine
Glibenclamide
Insulin Isophane
Insulin Mixtard
Insulin soluble
Metformin

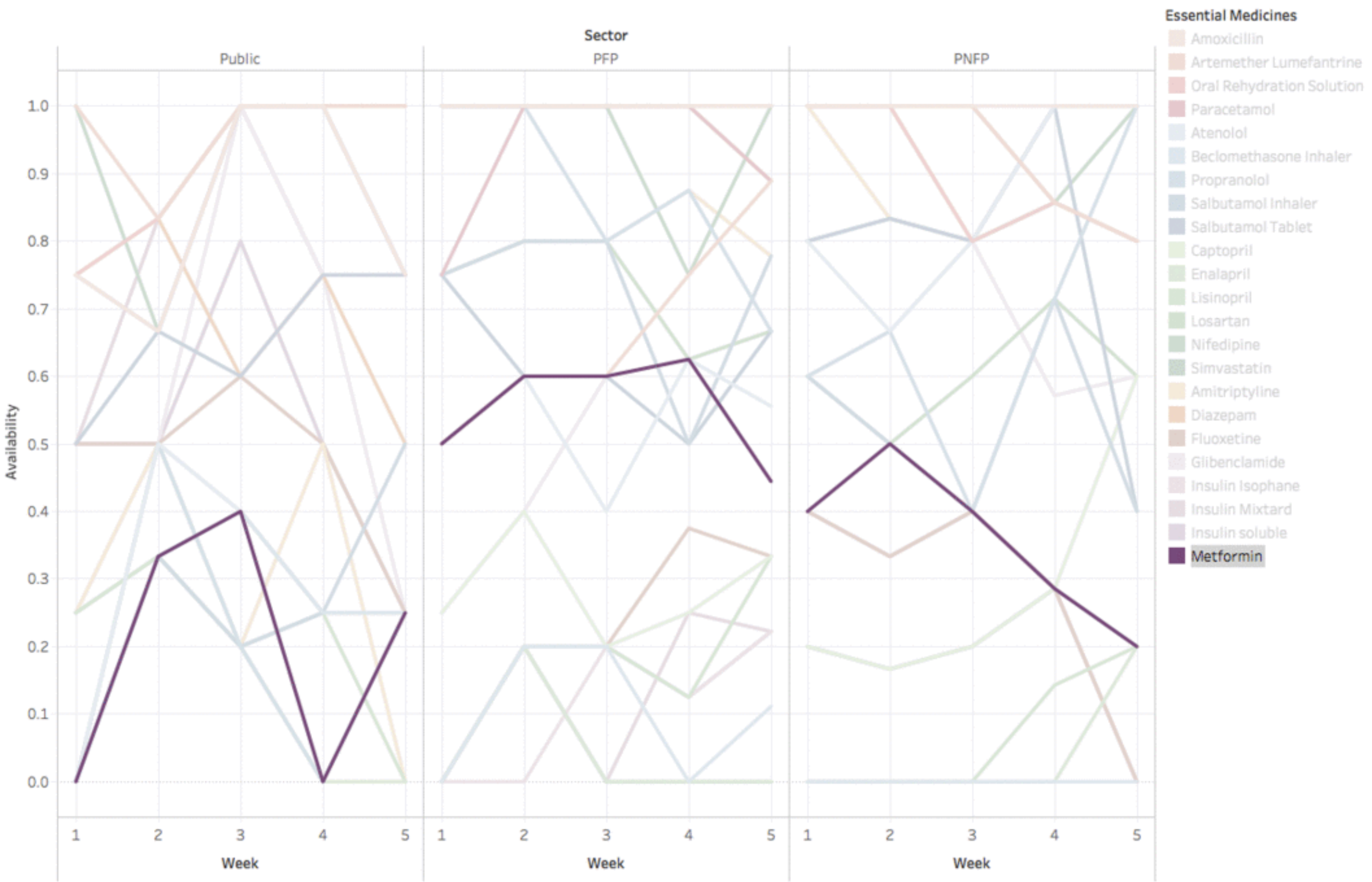
Sector
Public
PFP
PNFP
Availability
1.0
0.9
0.8
0.7
0.6
0.5
0.4
0.3
0.2
0.1
0.0
1
2
3
4
5
Week
Essential Medicines
Amoxicillin
Artemether Lumefantrine
Oral Rehydration Solution
Paracetamol
Atenolol
Beclomethasone Inhaler
Propranolol
Salbutamol Inhaler
Salbutamol Tablet
Captopril
Enalapril
Lisinopril
Losartan
Nifedipine
Simvastatin
Amitriptyline
Diazepam
Fluoxetine
Glibenclamide
Insulin Isophane
Insulin Mixtard
Insulin soluble
Metformin
